# Radical radiotherapy with concurrent weekly cisplatin in loco-regionally advanced squamous cell carcinoma of the head and neck: a single-institution experience

**DOI:** 10.1186/1758-3284-1-17

**Published:** 2009-06-15

**Authors:** Tejpal Gupta, Jai Prakash Agarwal, Sarbani Ghosh-Laskar, Purvish M Parikh, Anil K D'Cruz, Ketayun A Dinshaw

**Affiliations:** 1 Department of Radiation Oncology, ACTREC, Tata Memorial Centre, Kharghar, Navi Mumbai, India; 2 Department of Radiation Oncology, Tata Memorial Hospital, Mumbai, India; 3 Department of Medical Oncology, Tata Memorial Hospital, Mumbai, India; 4 Department of Surgical Oncology, Tata Memorial Hospital, Mumbai, India

## Abstract

**Background:**

The dominant pattern of failure for squamous cell carcinoma of head and neck remains loco-regional, although distant metastases are now being increasingly documented. Radical radiotherapy with concurrent chemotherapy is contemporary standard of care in the non-surgical management of these loco-regionally advanced cancers, based on large randomized controlled trials utilizing high-dose cisplatin (80–100 mg/m^2^) cycled every three-weekly during definitive radiotherapy. Although efficacious, this is associated with high acute morbidity necessitating intensive supportive care with attendant resource implications. The aim of this retrospective study was to assess the efficacy and acute toxicity of an alternative schedule i.e. concurrent weekly cisplatin-based radical radiotherapy and it's potential to be an optimal regimen in advanced head and neck cancers.

**Methods:**

Outcome data of patients with Stage III & IV head and neck squamous cell carcinoma, excluding nasopharynx, planned for radical radiotherapy (66–70 Gy) with concurrent weekly cisplatin (30 mg/m^2^) treated in a single unit between 1996–2004 was extracted.

**Results:**

The dataset consisted of 264 patients with a median age of 54 years. The median radiotherapy dose was 70 Gy (range 7.2–72 Gy) and median number of chemotherapy cycles was 6 (range 1–7). Two-thirds (65%) of patients received ≥85% of planned cisplatin dose. With a mean follow-up of 19 months, the 5-year local control; loco-regional control; and disease free survival was 57%; 46%; and 43% respectively. Acute grade 3 or worse mucositis and dermatitis was seen in 77 (29%) and 92 (35%) patients respectively, essentially in patients receiving doses ≥66 Gy and 6 or more cycles of chemotherapy. Other toxicities (hematologic, nausea and vomiting) were mild and self-limiting. Overall, the acute toxicity of this concurrent weekly chemo-radiation regimen though mildly increased did not mandate intensive supportive care. Stage grouping, primary site, and intensity of treatment were significant predictors of loco-regional control and disease free survival.

**Conclusion:**

Radical radiotherapy with concurrent weekly cisplatin has moderate efficacy and acceptable acute toxicity with potential to be an optimal regimen in loco-regionally advanced squamous cell carcinoma of the head and neck, particularly in limited-resource settings. Stage grouping, primary site, and treatment intensity are important determinants of outcome.

## Background

Squamous Cell Carcinoma of the Head and Neck (SCCHN) affects 550,000 new patients worldwide annually [1]. Traditionally surgery and radiotherapy (RT) either alone for early stage disease or in combination for loco-regionally advanced disease were considered to have curative potential [2,3]. Although the dominant presentation as well as pattern of failure for patients with SCCHN remains loco-regional, an increasing number of patients are being diagnosed with distant metastases [4]. The two most commonly employed strategies of improving outcome in SCCHN i.e. chemo-radiotherapy (CRT) and altered fractionation are attempts at treatment intensification. Ultimate treatment intensity is limited by patient-related, disease-specific, and environmental factors. Although intensification of treatment improves loco-regional control and consequently survival, it is associated with high acute morbidity necessitating intensive supportive care with resource implications. Recent advances in translational and clinical research have led to a paradigm shift wherein radical radiotherapy with concurrent chemotherapy (3-weekly high-dose cisplatin) is now considered the contemporary standard of care in the non-surgical management of loco-regionally advanced SCCHN [5-7]. Despite compelling evidence regarding the benefit of adding chemotherapy, there exists considerable difficulty in choosing the optimal CRT schedule due to heterogeneity of study designs and different ways of combining chemotherapy with RT [8].

This retrospective review attempts to analyze a large cohort of patients with loco-regionally advanced SCCHN treated uniformly with weekly cisplatin-based concurrent chemo-radiotherapy. Local control, loco-regional control, and disease-free-survival (DFS) were considered as measures of efficacy. Overall survival was not considered as an end-point, due to high non-cancer-related mortality secondary to the effects of ageing and prolonged tobacco and alcohol abuse, existent in this population.

## Aims and objectives

The primary aim of this study was to assess the efficacy and acute toxicity of radical radiotherapy with concurrent weekly cisplatin and help define an optimal CRT schedule with tolerable acute toxicity that could be administered on an outpatient basis to a large majority of patients with SCCHN without necessitating intensive supportive care in limited-resource settings. A secondary aim was to identify prognostic and therapeutic factors affecting outcome (local control, loco-regional control, and DFS) in patients with loco-regionally advanced SCCHN undergoing weekly cisplatin-based definitive concurrent CRT.

## Materials and methods

The medical records of all patients with SCCHN treated with radical RT in a single unit at the institute between 1996 and 2000 were reviewed retrospectively. A prospective SCCHN database maintained in the department was searched electronically from 2000 to 2004 to identify eligible patients. Newly diagnosed patients with loco-regionally advanced American Joint Committee on Cancer (AJCC) Stage III & IV SCCHN excluding nasopharyngeal cancers who were planned for definitive concurrent CRT based on a detailed assessment in a multi-disciplinary head and neck oncology joint clinic were included. Patients treated with neoadjuvant, adjuvant, or non-cisplatin based chemotherapy were excluded. Patients treated with conformal techniques were also considered ineligible due to potential confounding factors. A total of 264 patients were considered suitable and form the dataset for this analysis.

### Radiotherapy

Conventional RT was planned for all patients after appropriate immobilization using either a customized plaster of Paris cast or a thermoplastic mask. All patients were irradiated with megavoltage beams either on a telecobalt or a Linear Accelerator, with conventional fractionation (200 cGy per fraction, one fraction per day, 5 days per week) with shrinking field technique. The gross tumor volume was treated to a dose of 66–70 Gy in 33–35 fractions over 6.5–7 weeks. Areas of potential microscopic disease were treated till 50–60 Gy in 25–30 fractions over 5–6 weeks. Most patients were treated with bilateral opposing portals to the face and neck as per the institutional policy. Three-field technique (bilateral opposing for primary and upper neck matched onto a low anterior neck field) was used sparingly at the discretion of the treating oncologist. The radiation portals were dictated by primary site and disease stage. Beam modifiers and posterior neck boosts with appropriate electron energy were used as and when indicated. Spinal cord shielding was applied after 46 Gy in 23 fractions.

### Chemotherapy

Cisplatin (30 mg/m^2^) was administered intravenously concurrently weekly during the course of radiotherapy. Routine hydration with 500 mL normal saline given 30–45 minutes before chemotherapy and 1000 mL of normal saline given over 2 hours immediately after chemotherapy was used as per institutional guidelines. Standard anti-emetic prophylaxis consisted of 16 mg of ondansetron and 16 mg of dexamethasone given as intravenous bolus as pre-medication 30 minutes prior to chemotherapy. Anti-emetic prophylaxis was continued with ondansetron and domperidone or metoclopramide orally 2–3 days after each cycle of weekly cisplatin chemotherapy. Forced diuresis with 20% mannitol was at the discretion of the treating oncologist. Standard guidelines for dose reduction of cisplatin were applied by indirectly calculating the glomerular filtration rate based on Cockcroft-Gault formula.

The regimen was administered on an outpatient basis. All patients were monitored closely weekly during the course of CRT for assessing the toxicity of therapy. Toxicity grading was done according to the Radiation Therapy Oncology Group (RTOG) and Common Toxicity Criteria (CTC) grading systems for radiation-related and chemotherapy-related toxicities respectively. The patients were required to follow-up at 4–6 weeks from completion of therapy to assess response, toxicity and disease status. Subsequent follow-up visits were scheduled at 3–6 monthly intervals for the first 2 years and annually thereafter. At follow-up patients underwent thorough clinical examination for detection of loco-regional disease. Patients who dropped out or did not complete planned course of treatment were included as events for all the outcome measures. The disease status of patients who had completed the planned course of therapy, but not actively following-up was updated by telephonic contact. Non-responding patients were considered lost to follow-up and censored for statistical consideration.

### Statistical analysis

Local failure was defined as persistence of disease or reappearance of disease at or in close vicinity to the primary site. Loco-regional failure was defined as persistence of disease or reappearance of disease either at the primary site and/or draining regional lymph nodes. Relapse was defined as recurrence of disease at either the primary, regional or distant site or the appearance of a second primary in the upper aero-digestive tract. The local control, loco-regional control and DFS were calculated using the method of Kaplan-Meier. All estimates were calculated from the date of initiation of therapy till the defined event if any or until last contact or death. The analysis is limited to first failures only as a measure of efficacy of concurrent CRT and does not include outcome after attempted salvage for the stated endpoints. The data was compared using the log-rank test and Cox regression model for univariate and multivariate analysis respectively. All analysis was done on SPSS version 14.0 (Statistical Package for Social Sciences, IL, Chicago).

## Results

All patients with AJCC Stage III or IV SCCHN (excluding nasopharynx) planned for weekly cisplatin-based definitive concurrent CRT were included in the dataset. Patients receiving even one cycle of weekly cisplatin were considered eligible. Patients with progressive disease or dropouts after a few fractions of RT without completing the planned radical course either due to toxicity or socio-personal reasons were also included in the analysis to reduce bias inherent in retrospective analyses and as a reflection of ground realities in routine clinical practice.

### Clinical characteristics

The socio-demographic and clinico-pathologic characteristics of all the analyzable 264 patients with loco-regionally advanced SCCHN receiving radical RT with concurrent weekly cisplatin were consistent with previously published head and neck literature. The median age of the cohort was 54 years (range 20–79 years). The sex ratio was 5:1 in favor of males. Most of the patients had a good performance status with a median Karnofsky Performance Score (KPS) of 80 (range 60–100). Although medical co-morbidities consistent with the age-pyramid were prevalent, they were not significant enough in the large majority (such as active tuberculosis, uncontrolled hypertension or diabetes mellitus, or nephropathy) precluding systemic chemotherapy. The mean and median total dose of radiation for the entire cohort was 66 Gy and 70 Gy respectively (range 7.2–72 Gy). The median Overall Treatment Time (OTT) was 51 days with a range of 4–72 days. All patients received some concurrent cisplatin with the median number of weekly chemotherapy cycles being 6 (range 1–7). More than 65% (n = 169) patients received >85% of the planned dose of chemotherapy i.e. at least 6 cycles or more of concurrent weekly cisplatin (30 mg/m^2^).

### Efficacy analysis

**Figure 1:**
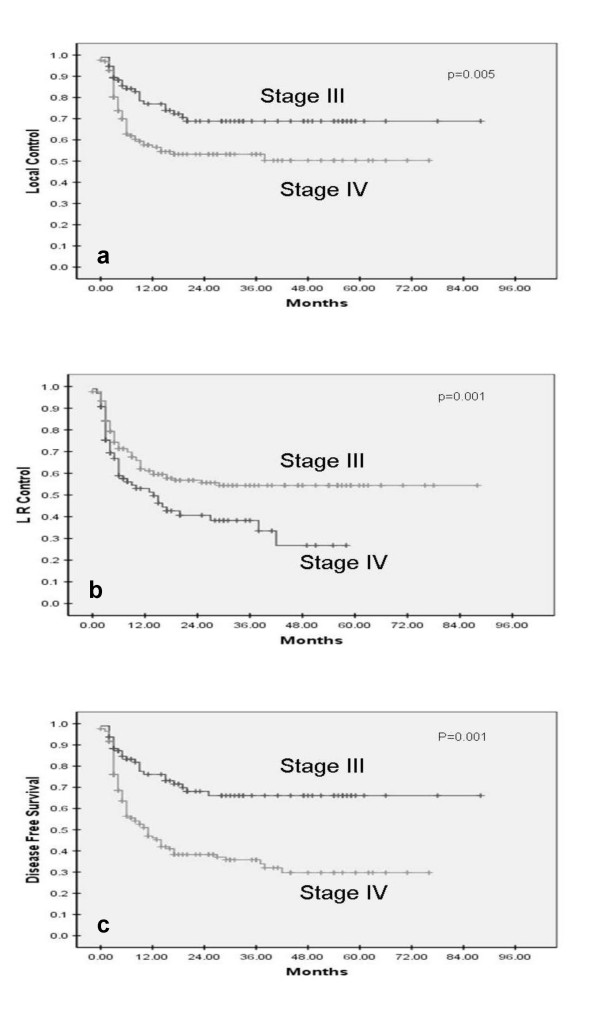
**Stage grouping as prognostic factor for local control (a), loco-regional control (b), and disease-free survival (c) in SCCHN treated with concurrent chemo-radiotherapy**.

With a mean follow-up of 17 months (range 0–88 months) for all patients and 19 months (range 0–88 months) for survivors, the 5-year local control; loco-regional control and DFS was 57.4%; 46.2%; and 43.3% respectively. Seven patients (2.7%) developed distant metastases and only 1 (0.4%) metachronous second primary tumor was detected on follow up. The impact of different prognostic factors on local control, loco-regional control, and DFS was then analyzed (Table 1). The AJCC stage grouping was highly significant in terms of all the outcome measures (Figure 1). The 5-year local control, loco-regional control and DFS was 68.8%, 66.1%, and 66.1% respectively for stage III disease as compared to 50.3%, 34%, and 29.7% for stage IV with highly significant p-values (p < 0.001). T-stage impacted significantly upon local control and showed some trend on loco-regional control too. N-stage did not affect local control, but had significant impact upon loco-regional control and DFS.

**Table 1:** Univariate analysis for local control, loco-regional control & disease free survival

**Prognostic factor**	**Patients**	**5-yr LC**	**p-value**	**5-yr LRC**	**p-value**	**5-yr DFS**	**p-value**
***Age***							
> 54 yrs	110	46.8%	0.057	36.2%	0.067	35.0%	0.123
≤ 54 yrs	154	65.1%		53.6%		49.0%	
***HP Grade***							
NOS	232	55.2%	0.115	44.2%	0.153	41.8%	0.241
WD	04	33.3%		33.0%		33.3%	
MD	05	80.0%		40.0%		40.0%	
PD	23	73.7%		64.2%		55.2%	
***T stage***							
T1-T2	53	73%	**0.004**	43.4%	0.079	36.1%	0.078
T3	153	59%		50.7%		49.3%	
T4	58	50%		36%		33.8%	
***N stage***							
N0	64	66.0%	0.212	66.0%	**0.001**	66.0%	**0.001**
N1	61	58.1%		54.1%		54.1%	
N2-3	139	51.9%		31.9%		27.0%	
***Stage Group***							
III	96	68.8%	**0.005**	66.1%	**0.001**	66.1%	**0.001**
IV	168	50.3%		34.0%		29.7%	
***Primary site***							
Oral cavity	18	13.9%	**0.001**	13.9%	**0.012**	13.9%	**0.026**
Oropharynx	118	58.6%		50.7%		47.7%	
Hypopharynx	93	59.3%		45.0%		42.9%	
Larynx	35	71.9		50.2%		44.4%	
***RT dose***							
< 70 Gy	51	51.2%	0.280	37.4%	0.125	35.9%	0.142
≥ 70 Gy	213	60.2%		50.5%		45.6%	
***CT cycles***							
1–5	96	41.8%	**0.022**	26.8%	**0.009**	25.8%	**0.011**
≥ 6	168	64.5%		54.4%		49.6%	
***KPS***							
< 80	51	46.4%	0.215	33.0%	0.183	30.3%	0.133
≥ 80	213	60.1%		49.3%		82.2%	
***OTT***							
≤ 51 days	116	67.7%	**0.029**	59.9%	**0.013**	56.5%	**0.016**
> 51 days	97	51.1%		39.3%		35.5%	

LC = local control; LRC = loco-regional control; DFS = disease free survival; NOS = not otherwise specified; WD = well differentiated; MD = moderately differentiated; PD = poorly differentiated; RT = radiotherapy; CT = chemotherapy; KPS = Karnofsky Performance Status; OTT = overall treatment time

**Figure 2:**
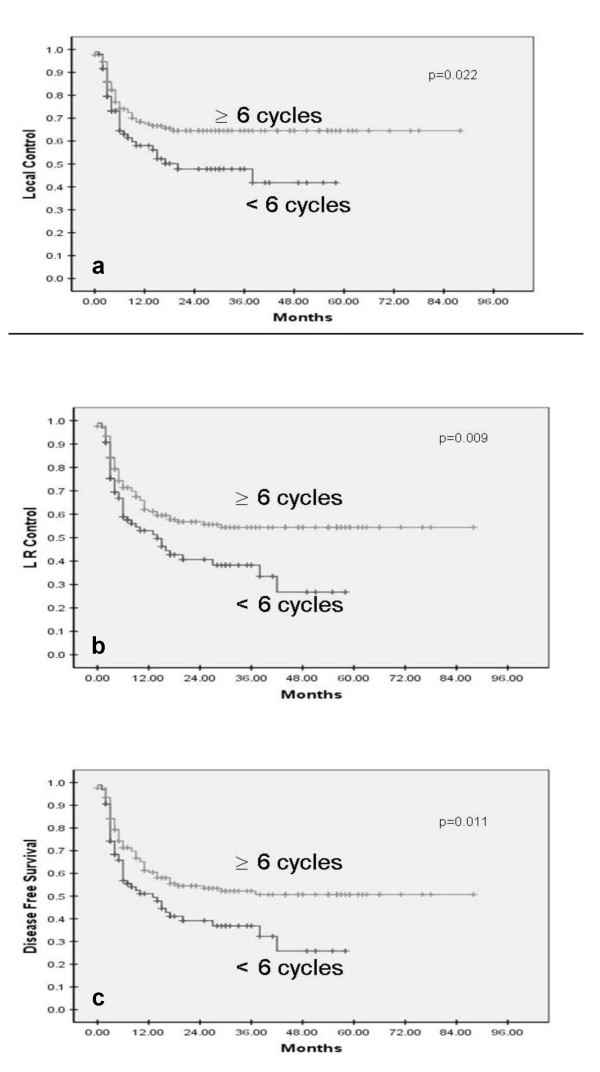
**Impact of intensity of chemotherapy on local control (a), loco-regional control (b), and disease-free survival (c) in weekly cisplatin-based concurrent chemo-radiotherapy for SCCHN**.

The intensity of treatment affected all three outcome measures significantly. Patients receiving >85% of the planned dose (6 or more cycles of weekly chemotherapy) had a significantly superior 5-year local control (64.5% vs 41.8%, p = 0.022); loco-regional control (54.4% vs 26.8%, p = 0.009); and DFS (49.6% vs 25.8%, p = 0.011) as compared to lesser dose intensity (1–5 cycles of chemotherapy) (Figure 2). On the basis of total dose of RT delivered, the patients were categorized into two dose groups, viz. <70 Gy; and ≥70 Gy. The 5-year local control in patients receiving <70 Gy was 51.2% as compared to 60.2% in patients receiving ≥70 Gy (p = 0.28). Similarly patients receiving higher total doses had better 5-year loco-regional control (50.5% vs 37.4%, p = 0.125) and DFS (45.6% vs 35.9%, p = 0.142). A separate analysis with 66 Gy as cut-off dose instead of 70 Gy also only showed some trend towards improved outcome with higher doses but was not statistically significant (data not shown). The reasons for choosing 66 and 70 Gy as the cut-off points was based on mean and median radiotherapy dose in this study cohort and previously published head and neck radiotherapy literature where radical doses have been defined as 66–70 Gy conventional fractionation equivalent. Overall treatment time (OTT) as a measure of treatment intensity was considered only for the 213 patients receiving at least 66 Gy. For this cohort, OTT was a significant predictor of outcome, with patients completing treatment in a shorter time (≤51 days) faring better overall than patients with longer OTT (>51 days). The 5-year local control (67.7% vs 51.1%, p = 0.029), loco-regional control (59.9% vs 39.3%, p = 0.013), and DFS (56.5% vs 35.5%, p = 0.016) was significantly better for patients completing optimal treatment within the planned time-frame. The cut-off for OTT was chosen at 51 days as it was the median of this cohort.

**Figure 3:**
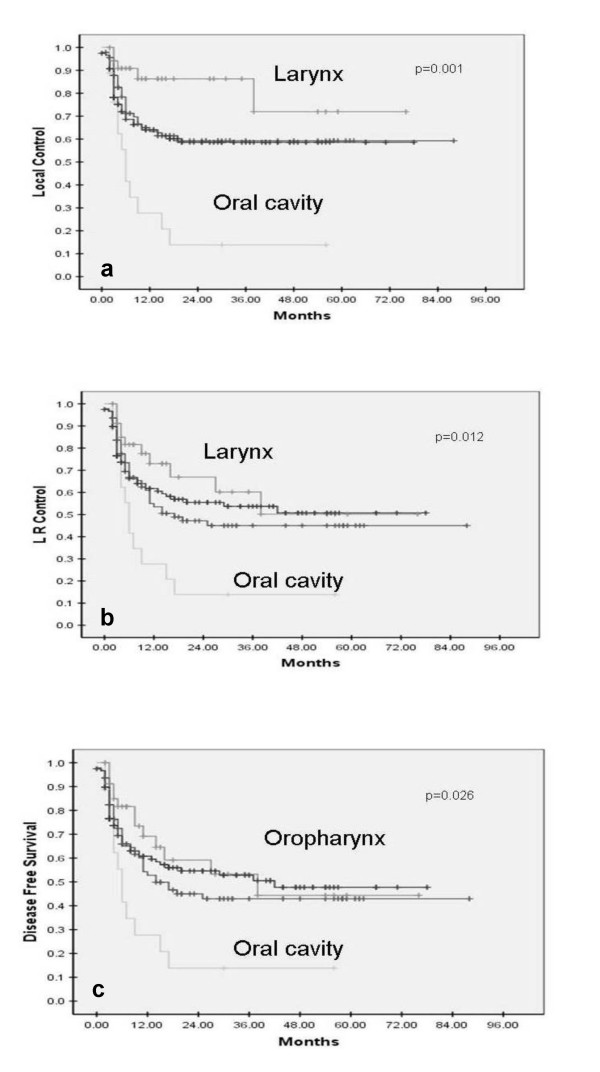
**Correlation of primary site with local control (a), loco-regional control (b), and disease-free survival (c) in loco-regionally advanced SCCHN treated with concurrent chemo-radiotherapy**.

Primary site was also a prognosticator of outcome (Figure 3). Patients with oral primaries fared the worst, with a 5-year local control, loco-regional control, and DFS of 13.9% each. In contrast patients with laryngeal and oropharyngeal cancers had the best 5-year outcomes. Their 5-year local control, loco-regional control and DFS was 71.9% & 58.6%; 50.2% & 50.7%; and 44.4% & 47.7% respectively. Hypopharyngeal primaries fared intermediately with 5-year outcomes of 59.3%, 45%, and 42.9% for local control, loco-regional control and disease free survival respectively. Oral cavity as a primary site has previously also been demonstrated as a poor prognostic factor, whereas larynx and oropharynx have done consistently better. A subset analysis of TNM stage grouping within each primary site re-inforced the importance of stage as the most significant determinant of outcome. The 5-year local control, loco-regional control and DFS were consistently and significantly better in Stage III as compared to Stage IV disease within each primary site (Table 2).

**Table 2:** Outcomes within each primary site correlated with AJCC stage

	**No. of patients**	**5-yr LC**	**p-value**	**5-yr LRC**	**p-value**	**5-yr DFS**	**p-value**
***Oral cavity***							
***Stage III***	2	50%	0.13	50%	0.13	50%	0.13
***Stage IV***	16	8.1%		8.1%		8.1%	
							
***Oropharynx***							
***Stage III***	40	68.8%	0.08	68.8%	**0.01**	68.8%	**0.004**
***Stage IV***	78	53.6%		36.4%		29.5%	
							
***Hypopharynx***							
***Stage III***	42	66.9%	0.16	59.3%	**0.01**	59.3%	**0.009**
***Stage IV***	51	53.2%		34.4%		31.2%	
							
***Larynx***							
***Stage III***	12	80.2%	0.83	80.2%	0.15	80.2%	0.07
***Stage IV***	23	67.8%		34.3%		28%	

LC = local control; LRC = loco-regional control; DFS = disease-free survival

Younger age as a prognostic factor did show a trend towards improved local and regional control which did not reach statistical significance (borderline significant p values). It is well-accepted that older patients tend to have worse outcomes with aggressive schedules due to lower compliance and higher toxicity rates. Patients with good KPS also tended to do better, although once again it was not statistically significant. Histological grade and sex were not found to affect outcome significantly. All factors that were of significance or of borderline significance on univariate analysis were considered for multivariate analysis. Multivariate analysis using Cox proportional hazards model confirmed the importance of AJCC stage grouping, total chemotherapy dose, and primary site as significant and independent prognostic factors (Table 3).

**Table 3:** Significant factors in multivariate analysis for outcome measures

**Prognostic factor**	**p-value**	**Hazard Ratio**	**95% Confidence Interval**
***Local Control***			
Stage grouping (III vs IV)	0.013	1.816	1.134 – 2.908
Primary site (oral vs non-oral)	0.001	0.648	0.497 – 0.845
No of CT cycles (less vs more)	0.038	0.643	0.424 – 0.977
***Loco-regional Control***			
Stage grouping (III vs IV)	0.000	2.406	1.551 – 3.734
No of CT cycles (less vs more)	0.014	0.627	0.432 – 0.909
***Disease Free Survival***			
Stage grouping (III vs IV)	0.000	2.640	1.707 – 4.081
No of CT cycles (less vs more)	0.016	0.642	0.447 – 0.922

### Toxicity analysis

The RTOG acute grade 3 or worse mucositis and dermatitis was seen in 77 (29.2%) and 92 (34.8%) patients respectively, most of the time in patients receiving more intense treatment i.e. doses ≥66 Gy and 6 or more cycles of chemotherapy. Mild to moderate nausea and vomiting occurred in almost all patients despite anti-emetic prophylaxis. CTC grade 3 or worse emesis occurred in 9 (3.4%) patients, mostly towards the end of chemo-radiotherapy. Acute hematologic toxicity in the form of leucopenia and thrombocytopenia was mild and acceptable. The incidence of CTC grade 3 leucopenia was 5.7%. No episodes of febrile neutropenia were recorded. No patients experienced CTC grade 3 thrombocytopenia. Platelet transfusion or growth factor support due to acute hematologic toxicity was not needed in any patient. There was minimal acute kidney dysfunction, with no episodes of grade 3 or worse renal toxicity because the dose of cisplatin was titrated based on indirect estimation of glomerular filtration rate prior to each cycle of weekly chemotherapy. Toxicity of treatment leading to interruption or compromise in the planned dose of radiotherapy was seen in 40 (15%) patients. Twenty (7.5%) patients had to be hospitalized for supportive care. Only 5 (2%) patients dropped out of treatment and did not complete even 50 Gy of RT, mostly due to social and personal reasons and not due to toxicity. Overall the regimen was well tolerated with acceptable acute toxicity. An analysis of late effects of the regimen was not attempted due to lack of proper documentation of late toxicity.

Salvage treatment at relapse was individualized and at the discretion of the treating oncologist. The majority of patients (42/68) with relapse were offered best supportive care alone. Salvage surgery was attempted in 22 patients, 6 of whom received adjuvant re-irradiation based on surgical pathology. Palliative chemotherapy with or without re-irradiation was considered appropriate for 4 patients. However since very few patients were effectively salvaged, this analysis did not attempt to consider the impact of salvage therapy on final outcome restricting itself to first failures alone, a measure of the efficacy of radical chemo-radiotherapy as definitive treatment.

## Discussion

This study represents one of the largest single-centre experiences of weekly cisplatin concurrently with radiotherapy in the non-surgical management of loco-regionally advanced SCCHN. Patients who were offered upfront surgery followed by adjuvant chemo-radiotherapy were not considered for this study and constitute the dataset of a separate report. This study confirms the importance of AJCC stage grouping, intensity of treatment, and primary site as significant predictors of outcome, consistent with published head and neck literature.

The most robust evidence recommending platinum-based concurrent CRT as the standard of care for loco-regionally advanced SCCHN comes form rigorously conducted randomized controlled trials and meta-analyses [5-7] using mortality as outcome of interest. The updated meta-analysis of chemotherapy in head and neck cancer (MACH-NC) confirmed the findings of the original report of 4% overall survival benefit with chemotherapy [6,7]. It also demonstrated a relative 19% improvement in survival (hazard ratio 0.81; p < 0.001) for concomitant therapy translating into an 8% absolute benefit in overall survival with platinum-based regimens in the concurrent setting. However, little is known about the extent to which concurrent CRT for SCCHN has been adopted in the community setting and whether benefits commensurate with those reported in trial setting are achieved in community practice [8,9]. Despite robust evidence and consistent guidelines [10,11], uncertainties exist regarding appropriate CRT regimens because of significant heterogeneity in published data with respect to patient selection, chemotherapy schedules and RT fractionation [8,9]. RT still remains the primary and definitive component of concurrent chemo-radiation. Therefore, it is imperative that concurrent chemotherapy be integrated into the same radiation regimen that would constitute optimal treatment if radiotherapy was administered as mono-therapy. A fundamental corollary is that a concurrent CRT regimen proven superior only to a sub-optimal radiation alone regimen does not constitute optimal therapy [12]. The increased efficacy of CRT needs to be carefully balanced against the enhanced acute and perhaps even long-term toxicity. Enhanced acute toxicity leading to interruption or modification of radiation delivery may potentially compromise on the efficacy of treatment. Intensive supportive care with liberal use of feeding tubes, intravenous hydration and narcotic analgesics may be a challenge even for seasoned health-care providers [13] with attendant resource implications.

The putative mechanisms of synergistic interaction of cisplatin with radiotherapy in SCCHN include radiosensitizer (through inhibition of potentially lethal damage repair and sublethal damage repair); hypoxic cell sensitizer; cell cycle pertubator; ability to form deoxyribonucleic acid (DNA) adducts; and inhibition of angiogenesis [14]. Concurrent 3-weekly high-dose cisplatin (100 mg/m^2^) is contemporary 'standard of care' for loco-regionally advanced SCCHN based on level I evidence [5,6]. However, only 60% of patients in clinical trial setting are able to receive all the three planned doses of three-weekly cisplatin due to unacceptably high systemic and mucosal toxicities [12,15]. The lack of uniform reporting of side effects and small size of individual studies limits conclusions about the relative tolerability of one regimen over the other. Schedules that deliver smaller doses at more frequent intervals are also quite effective in improving outcome. More frequent administration could provide better radio-sensitization to a larger proportion of the administered radiotherapy dose [12]. Marcu and colleagues [16] studied the scheduling of cisplatin with radiotherapy in a previously developed tumor growth model of SCCHN by implementing the kinetics of cisplatin and concluded that daily low dose immediately prior to each fraction of radiation could result in best radiosensitization. Smaller individual doses of the drug may also result in lesser chemotherapy induced morbidity without compromising the efficacy. Although no phase I data for weekly scheduling of cisplatin for SCCHN existed, the dose-intensity of prevailing regimens was in the range of 25–33 mg/m^2 ^per week. Similar weekly regimen was being offered to patients with carcinoma cervix with radical radiotherapy as an institutional policy. There are now several reports showing benefit in loco-regional control and/or survival with alternative cisplatin regimens, i.e. 5 doses of 20 mg/m^2 ^for 5 consecutive days [17] or 4 doses of 25 mg/m^2 ^for 4 consecutive days [18] during weeks 1, 4, and 7 of radiotherapy; weekly doses of 40–60 mg for 6–7 weeks [19,20]; and 5–7 mg/m^2^/day, 5 days a week during a 7 week course of fractionated radiotherapy [21-23].

The uncertainty regarding the optimal scheduling of cisplatin with radiotherapy in SCCHN has sparked considerable interest in comparing various dose-schedules. In the first such comparative study [24], 51 patients received cisplatin (100 mg/m^2 ^over 2–3 days) during weeks 1, 4, and 7, while 32 patients received cisplatin (40 mg/m^2^) weekly during definitive radiotherapy. Contrary to expectations, the incidence of severe acute toxicity (skin, hematological, treatment interruptions, weight loss, and mucositis) was significantly higher (p = 0.005) in the weekly cisplatin arm, prompting the authors to suggest chemotherapy dose reduction and prophylactic feeding tube placements in the weekly arm. In another indirect comparison, Ho and colleagues [25], compared the differences in dose intensity, delays, and toxicity between concurrent 3-weekly (80–100 mg/m^2^) and weekly (40 mg/m^2^) cisplatin-based definitive CRT in 51 patients with advanced SCCHN. More patients received a higher cumulative dose of at least 240 mg/m^2 ^in the weekly arm as compared to the 3-weekly arm (p = 0.04). The 3-weekly regimen was associated with more delays (41% vs 29%) and omissions of chemotherapy (17.4% vs 5.6%) resulting in lesser patients achieving cumulative doses beyond 200 mg/m^2^, potentially lowering dose-intensity. A prospective non-randomized study [26] compared 3-weekly cisplatin (100 mg/m^2^) given to younger patients with good KPS (n = 30) with weekly cisplatin (40 mg/m^2^) in patients with older age or poor KPS (n = 20) along with radical radiotherapy. The complete response rate (50% vs 40%), overall response rate (92% vs 90%), and grade III-IV toxicities (53% vs 40%) were similar in the two cohorts. The only randomized study comparing daily (6 mg/m^2^), weekly (40 mg/m^2^), and three-weekly (100 mg/m^2^) schedule of cisplatin with conventionally fractionated radiotherapy [27] did not find any significant difference in the efficacy of the regimens (similar response rates and loco-regional control), but reported varying degrees of mucosal, renal and hematologic toxicity. Overall the available data suggests that a cumulative cisplatin dose of 200–250 mg/m^2 ^given three-weekly, weekly, or daily during radiotherapy yields therapeutic benefit [12,15].

The most popular schedule of concurrent cisplatin for SCCHN outside the context of clinical trials is not the three-weekly regimen but a weekly schedule [28] of cisplatin in the dose range of 30–40 mg/m^2^. Most of the bias against using weekly cisplatin stems from the negative results of a single inter-group trial [29] which has been reported only in abstract form. There was no difference in outcome for 319 evaluable patients randomized to concurrent weekly cisplatin plus conventionally fractionated radiotherapy versus radical radiotherapy alone. However, approximately 15% of patients in the trial were nasopharyngeal cancers and the final results have never been published in full form. Although additional data from the trial was made available for the MACH-NC analysis, it is inadequate to draw definitive conclusions regarding the efficacy of weekly cisplatin. In a more recent phase III trial [30], involving 153 stage II-IV oropharyngeal and nasopharyngeal cancer patients, Sharma et al reported improved response rates (79.2% vs 69.7%, p < 0.05) and 3-year overall survival (62% vs 42%, p = 0.024) for concurrent weekly cisplatin as compared to radical radiotherapy alone. This however, was achieved at the cost of increased grade III-IV toxicities (40% vs 16%, p < 0.05), more frequent interruptions (28.9% vs 9.3%, p < 0.05), and hospitalization (40.8% vs 20%, p < 0.05), prompting the authors to conclude that the enhanced toxicity of concurrent chemoradiation remains an area of concern for the constrained medical infrastructure of a developing country economy.

Some of the current generation co-operative group trials are also using weekly chemotherapy with single agent carboplatin with or without taxanes in the concurrent setting for loco-regionally advanced SCCHN [15], either as definitive treatment or after induction chemotherapy.

Recent evidence supports the use of altered fractionation for improvement in outcome for SCCHN [31]. Concurrent chemotherapy with altered fractionation has the potential to improve outcomes significantly [18,19] albeit at the cost of substantially increased acute and late toxicity [32,33]. Since the toxicity of weekly cisplatin in the given dose range is substantially lower than the high-dose three-weekly schedules, combining weekly chemotherapy with altered fractionation may be more acceptable to the practicing oncologist.

## Conclusion

This study reports on one of the largest single-centre experience of weekly cisplatin concurrently with radiotherapy with potential to be an optimal therapeutic regimen in the non-surgical management of loco-regionally advanced SCCHN. AJCC stage grouping, intensity of treatment, and primary site were significant and independent predictors of outcome. The efficacy of this regimen is largely comparable to other contemporary series. Acute toxicity is substantially lower than previously reported for more intensive CRT schedules. There is significant scope for further optimizing cisplatin-based concurrent chemoradiotherapy regimens through rigorous pharmacokinetic and radiobiologic modeling. Larger prospective trials exploring various schedules of cisplatin (dose, route, frequency, and sequence) are warranted in order to find the most optimal way of combining the drug with radiation.

## Abbreviations

SCCHN: squamous cell carcinoma of the head and neck; RT: radiotherapy; CRT: chemoradiotherapy; DFS: disease free survival; AJCC: American Joint Committee on Cancer; RTOG: Radiation Therapy Oncology Group; CTC: Common Toxicity Criteria; SPSS: Statistical Package for Social Sciences; KPS: Karnofsky performance status; OTT: overall treatment time; MACH-NC: Meta-amalyses of chemotherapy in head neck cancer; DNA: deoxyribonucleic acid.

## Competing interests

The authors declare that they have no competing interests.

## Authors' contributions

JPA, PMP, and AKD conceived the study. TG, SGL, and JPA reviewed the case records and extracted the data. TG and JPA did data analysis and interpretation. TG and SGL wrote the initial draft and revised the manuscript. KAD, JPA, AKD critically reviewed the manuscript and approved final version.
